# Establishment of a prognostic risk model for osteosarcoma and mechanistic investigation

**DOI:** 10.3389/fphar.2024.1399625

**Published:** 2024-04-24

**Authors:** Hongyuan Jiang, Xuliang Zhao, Jinhui Zang, Ruijiao Wang, Jiake Gao, Jinli Chen, Tengbo Yu

**Affiliations:** ^1^ Department of Sports Medicine, Affiliated Hospital of Qingdao University, Qingdao, Shandong, China; ^2^ Qingdao Medical School, Qingdao University, Qingdao, Shandong, China; ^3^ Qingdao Municipal Hospital, Qingdao, Shandong, China

**Keywords:** immunology, osteosarcoma, prognosis diagnosis model, CHMP4C, GSK3β/βcatenin signaling pathway

## Abstract

**Objective:** To investigate the immune mechanism of osteosarcoma (OS)-specific markers to mitigate bone destruction in the aggressive OS, prone to recurrence and metastasis.

**Methods:** Gene expression patterns from the Gene Expression Omnibus (GEO) database (GSE126209) were analyzed using weighted gene co-expression network analysis (WGCNA), protein-protein interaction (PPI) analysis, least absolute shrinkage and selection operator (LASSO) modeling, and survival analysis to identify charged multivesicular body protein 4C (CHMP4C). Subsequently, its role in regulating the immune system and immune cell infiltration was explored. CHMP4C expression and signaling molecules in OS were assessed in osteosarcoma cell lines (MG63, U2OS, HOS) and hFOB1.19 cells using reverse transcription-quantitative polymerase chain reaction (RT-qPCR) and immunofluorescence staining. The impact of CHMP4C upregulation and interference on OS-related signaling molecules in MG63 cells was studied. Functional validation of CHMP4C in MG63 OS cells was confirmed through cell counting Kit-8 (CCK-8), transwell, and colony formation assays. *In vivo* experiments were conducted using Specific Pathogen Free (SPF)-grade male BALB/C nude mice for OS xenograft studies.

**Results:** Based on the gene expression profiles analysis of six osteosarcoma samples and six normal tissue samples, we identified 1,511 upregulated DEGs and 5,678 downregulated DEGs in normal tissue samples. A significant positive correlation between the “yellow-green” module and OS was found through WGCNA analysis. Expression levels of CHMP4C, phosphorylated Glycogen Synthase Kinase 3β (p-GSK3β), and β-catenin were notably higher in U2OS, HOS, and MG63 OS cells than in hFOB1.19 human osteoblasts. Overexpressing CHMP4C in MG63 OS cells upregulated CHMP4C, p-GSK3β, and β-catenin while downregulating GSK3β, leading to increased proliferation and migration of MG63 cells. Conversely, interrupting CHMP4C had the opposite effect. High expression of CHMP4C significantly accelerated the growth of OS in nude mice, resulting in substantial upregulation of CHMP4C, p-GSK3β, and β-catenin expression and suppression of Glycogen Synthase Kinase 3β (GSK3β) expression in OS tissues.

**Conclusion:** CHMP4C may serve as a specific immunomodulatory gene for OS. Its activation of the Wnt/β-catenin signaling pathway, mainly by increasing the phosphorylation echelon of GSK3β, promotes the invasion and spread of OS.

## 1 Introduction

Osteosarcoma (OS) represents a prevalent malignant bone neoplasm, predominantly manifesting in the adolescent demographic. It is characterized by high malignancy, rapid growth, and a tendency to lead to serious issues such as lung metastasis, resulting in poor prognosis (Gianferante et al., 2017). The etiology of OS is substantially influenced by environmental, and epidemiological factors, and genetic damage (Zhao et al., 2014). OS primarily targets the metaphyseal extremities of long bones, including the distal femur, proximal tibia, and proximal humerus, displaying a predilection for distant metastasis, notably to the lungs. The 5-year survival rate for individuals with primary OS fluctuates between 65% and 70%, whereas for those with metastatic OS, it dwindles to a range of 19%–30% (Meyers et al., 2005). Contemporary strategies for managing OS primarily encompass surgical procedures, chemotherapy (both neoadjuvant and adjuvant), radiation therapy, immunotherapy, targeted therapy, and various other modalities (Wu et al., 2016). Despite continuous efforts to explore the pathogenesis of OS and develop new treatment strategies, treatment outcomes remain suboptimal, and overall survival rates have not significantly improved. Therefore, further research is required to elucidate the characteristics of OS, identify effective prognostic markers for accurate prognosis prediction, and select more proactive intervention measures. Unearthing novel prognostic markers holds the potential for advancing innovative treatment modalities.

Gene therapy has surfaced as a promising avenue in the OS treatment landscape, drawing considerable focus in the quest for efficacious therapeutic targets. Anomalous activation of the Wnt/β-catenin signaling pathway is a pivotal player in shaping the biological demeanor of OS. Invasion and migration stand as cardinal traits of OS cells. Besides, the Wnt/β-catenin signaling pathway can expedite pulmonary metastasis, exacerbating the disease by heightening the invasion and migration propensities of OS cells (Wang et al., 2021). Some studies have explored the mechanism by which this pathway functions by inhibiting the Wnt/β-catenin signaling pathway. For example, the Wnt receptor antagonist Dickkopf-related protein 3 (DKK-3) can reduce the invasive ability of OS cells such as SaOS2 cells (Baker et al., 2015). Key molecules in the Wnt/β-catenin signaling pathway include glycogen synthase kinase 3β (GSK3β), phosphorylated glycogen synthase kinase 3β (p-GSK3β), and β-catenin (Hagiwara et al., 2017). Increased nuclear β-catenin content can promote epithelial-mesenchymal transition in OS cells, enhance stem cell formation, and accelerate the invasion and migration of OS cells (Yu et al., 2020). GSK3β serves as a versatile serine/threonine protein kinase, wielding a pivotal influence on the transcription, proliferation, and apoptosis of tumor cells. Its involvement in phosphorylating β-catenin can instigate the activation of E3 ubiquitin ligase subunit β-Trcp, orchestrating the targeting of β-catenin for proteasomal degradation (Li et al., 2017). Moreover, phosphorylation of GSK3β induces its inactivation, triggering the buildup of β-catenin and facilitating its translocation into the cellular nucleus (MacDonald et al., 2009). Hence, GSK3β operates as a negative modulator within the Wnt/β-catenin signaling pathway. While certain studies suggest GSK3β and β-catenin as conceivable therapeutic targets for clinical management of OS, there remains a critical imperative to pinpoint novel diagnostic and prognostic biomarkers, enhancing precision in OS diagnosis and survival prognostication.

In this study, we employed bioinformatic methods to analyze critical gene expression differences between OS and normal tissues using data from the Gene Expression Omnibus (GEO) database. We used weighted gene co-expression network analysis (WGCNA) to identify gene modules associated with the clinical features of OS. Through least absolute shrinkage and selection operator (LASSO) analysis, we selected potential prognostic genes and constructed a model. In conclusion, we validated a notable association between charged multivesicular body protein 4C (CHMP4C) gene expression and the prognosis of individuals with OS. Furthermore, we delved into the biological functionalities of CHMP4C within OS cells and mouse xenografts of OS, aiming to dissect the potential mechanisms underpinning CHMP4C’s operation and discern its plausible role as a therapeutic target in the context of OS.

## 2 Materials and methods

### 2.1 Differential gene expression analysis

We performed differential gene analysis between osteosarcoma and normal tissue samples using DEseq2 in R version 4.0.1 (Lucent Technologies Inc., Union, NJ, USA, https://www.r-project.org/). The samples were labeled as “normal” and “tumor.” We calculated the fold change (FC) for genes and subsequently filtered the differentially expressed genes (DEGs) based on the following criteria: adjusted *p* < 0.05, log_2_FC > 1, or log_2_FC < −1. Subsequently, we used the ggplot package (https://cran.rproject.org/web/packages/ggplot2/index.html) and the ‘pheatmap’ package (https://cran.r-project.org/web/packages/pheatmap/index.html) in R to generate volcano plots and heatmaps. Additionally, we performed Gene Ontology (GO) annotation and Kyoto Encyclopedia of Genes and Genomes (KEGG) pathway enrichment analysis for the DEGs with the ‘clusterProfiler’ package in R version 4.0.1, with a significance threshold set at a *p*-value below 0.05 (Lei et al., 2015).

### 2.2 Database information retrieval

This study extracted osteosarcoma gene microarray data, including gene expression profiles from six osteosarcoma samples and six normal tissue samples, from the GEO database (https://www.ncbi.nlm.nih.gov/geo/query/acc.cgi?acc=GSE126209) at the National Center for Biotechnology Information (NCBI) in the United States.

### 2.3 Weighted gene co-expression network analysis (WGCNA)

We utilized the WGCNA analysis package in R version 4.0.1, with the aim of identifying gene modules that are highly correlated with osteosarcoma (Horvath and Dong, 2008). We computed scale independence (R2) and average connectivity to determine the soft threshold that conforms to an unscaled distribution, which was used for feature gene selection. Following this, we utilized hierarchical clustering and dynamic tree-cutting methods to discern gene modules, employing a substantial merging threshold of 0.25 to amalgamate akin gene modules. Ultimately, we computed both gene significance (GS) and module significance (MS) to evaluate the correlation and significance of genes in relation to clinical information.

### 2.4 Protein-protein interaction analysis (PPI)

We used the STRING website (https://string-db.org/) to perform PPI analysis on all genes within the green-yellow module. Subsequently, we conducted further analysis and visualization using the Molecular Complex Detection (MCODE) plugin in the Cytoscape software (http://cytoscape.org/).

### 2.5 Prognostic model construction and validation

In this study, we utilized the ‘glmnet’ package in R for LASSO regression analysis to screen core genes within the ‘greenyellow’ module (Park et al., 2015). LASSO regression analysis identifies variables by detecting the optimal λ value that minimizes classification error. The optimal value of the penalty parameter λ was selected using tenfold cross-validation. Receiver Operator Characteristic (ROC) curves were generated for predictive models via ‘timeROC’ package in R. The Area Under the Curve (AUC) was computed to gauge the predictive efficacy of the prognostic gene model. All statistical analyses were carried out through R software, with significance determined at *p* < 0.05 for all tests. Optimal cutoff points for risk scores and survival time were determined using the ‘maxstat’ package in R, and the training dataset was stratified into high-risk and low-risk groups based on these cutoff points. We utilized Kaplan-Meier analysis to compare overall survival times between the high-risk and low-risk groups. Survival curves for these groups were constructed with the ‘survminer’ and ‘survival’ packages in R, and inter-group differences were evaluated through chi-square testing.

### 2.6 Cell cultivation

The cell lines used in this study included osteosarcoma cells (MG63 cells, catalog number CL-0157; U2OS cells, catalog number CL-0236; HOS cells, catalog number CL-0360) and human fetal osteoblast hFOB1.19 cells (CL-0353), all of which were purchased from Procell Technology Co., Ltd. (China). These cells were centrifuged during cultivation and then supplemented with 1 mL of Dulbecco’s Modified Eagle Medium (DMEM) medium (C11995500BT, GIBCO, Thermo Fisher Scientific, Inc., USA) containing 10% fetal bovine serum (FBS) (Peak Inc., USA), 1% penicillin-streptomycin (Beyotime Institute of Biotechnology, China), and 20 ng/mL epidermal growth factor (EGF) (catalog number P00033, Solarbio Science & Technology Co., Ltd., China). All cells were cultured at 37°C in a 5% CO_2_ incubator.

### 2.7 Reverse transcription-quantitative polymerase chain reaction (RT-qPCR)

According to the reagent kit instructions, TRIzol reagent (Thermo Fisher Scientific, Inc.) was used to extract total RNA (Wang et al., 2020). Subsequently, we employed Tsingke Biotechnology Co., Ltd.'s Goldenstar™ RT6 cDNA Synthesis Kit Ver.2 (TSK302M, China) for reverse transcription, with the reaction conditions set at 25°C for 10 min, 55°C for 30 min, and 85°C for 5 min. To assess mRNA expression levels, we used the 2×T5 Fast RT-qPCR Mix (SYBR Green I) reagent kit from Tsingke Biotechnology Co., Ltd. (TSE002, China), with reaction conditions of 95°C for 30 s, followed by 40 cycles of 95°C for 3 s and 60°C for 30 s. We selected human glyceraldehyde-3-phosphate dehydrogenase (GAPDH) as the reference gene and calculated relative gene expression levels using the 2^−ΔΔCt^ method. Specific primer information for RT-qPCR is presented in Table 1.

**TABLE 1:** Primer sequences.

Primers	Sequences
CHMP4C-F	CCT​GGA​GAA​CTC​ACA​CAC​CA
CHMP4C-R	GTC​ATC​ACC​AAA​GCC​AAC​CC
GSK3β-F	GGA​CTA​AGG​TCT​TCC​GAC​CC
GSK3β-R	TGG​CAT​TTG​TGG​GGG​TTG​A
β-catenin-F	ATG​ATG​GTC​TGC​CAA​GTG​GG
β-catenin-R	GGC​CAT​CTC​TGC​TTC​TTG​GT
h-GAPDH-F	TCA​AGG​CTG​AGA​ACG​GGA​AG
h-GAPDH-R	TCG​CCC​CAC​TTG​ATT​TTG​GA

### 2.8 Immunofluorescence assay

In fixed cell cultures, 0.3% Triton X-100 (ST795, Beyotime Institute of Biotechnology, China) was added and incubated at 37°C for 5 min. Goat serum (C0265, Beyotime Institute of Biotechnology, China) was then added and incubated at room temperature for 60 min. Subsequently, the sections were incubated with antibodies against β-catenin (A19657, ABclonal Technology, China), CHMP4C (bs-7744R, Bioss Biotechnology Co., Ltd., China), GSK3β (A2081, ABclonal Technology, China), and p-GSK3β (bs-3161R, Bioss Biotechnology Co., Ltd., China) overnight at 4°C. After washing with Phosphate-Buffered Saline (PBS) (G0002, Sevier Biotechnology Co., Ltd., China), FITC Goat Anti-Rabbit IgG (H + L) (AS024, ABclonal Technology, China) was applied and incubated in the dark at 25°C for 1.5 h. Sections were subjected to 4′,6-diamidino-2-phenylindole (DAPI) staining (C1005, Beyotime Institute of Biotechnology, China), followed by a 5-min incubation in the dark and removal of excess DAPI through PBS wash. Subsequently, these sections were sealed with an anti-fade mounting medium (P0126; Beyotime Institute of Biotechnology, China). Ultimately, an inverted fluorescence microscope (ICX41; Sunny Optical Technology (Group) Co., Ltd., China) was employed to observe and capture images of the sections.

### 2.9 CHMP4C gene overexpression and lentivirus construction and packaging

The lentiviral overexpression vector pLVX-IRES-puro-CHMP4C and interference vector pLVX-shRNA1-CHMP4C were constructed by Chongqing Biomedicine Co., Ltd. (China). MG63 cells were passaged at a density of 1×10^5^ cells/well in a 24-well plate. After 24 h of incubation with fresh culture medium, the control group received 30 μL of empty viral suspension and 1 μL of polybrene (ZY140621, ZeYe Biotechnology Co., Ltd., China), whereas the experimental group received 30 μL of either overexpression lentivirus or interference lentivirus suspension and 1 μL of polybrene. The cells were then placed in a cell culture incubator for 48 h. Subsequently, the cells were continuously passaged, and the culture medium was changed every 2 days and supplemented with 5 μg/mL puromycin (ST551-10, Beyotime Institute of Biotechnology, China) for selection to obtain CHMP4C overexpressing MG63 cells (OE-CHMP4C), CHMP4C interference MG63 cells (IN-CHMP4C), and their corresponding empty vector control MG63 cells (OE-NC, IN-NC).

### 2.10 Cell counting Kit-8 (CCK-8) analysis

Cells in the logarithmic growth phase were harvested and plated into 96-well plates. Following that, 10 μL of CCK-8 solution (C0038; Beyotime Institute of Biotechnology, China) was introduced to each well. After a 1-h incubation at 37°C in a cell culture incubator, the absorbance of each well was assessed at 450 nm with a microplate reader (CMax Plus, Molecular Devices Instruments Co., Ltd., USA).

Cell viability (100%) was calculated as follows:

Experimental group: Absorbance of cells overexpressing or silencing plasmids along with CCK-8 solution.

Blank group: Absorbance of wells containing culture medium and CCK-8 solution without cells.

Control group: Absorbance of wells containing empty vector-transfected cells and CCK-8 solution.

$$\text{Cell viability }\left(100\%\right)=\left(\text{experimental group}–\text{blank group}\right)/\left(\text{control group}–\text{blank group}\right)\times 100$$

### 2.11 Plate cloning

Logarithmically growing CHMP4C-overexpressing MG63 cells, CHMP4C-knockdown MG63 cells, and their respective empty vector control cells were harvested to prepare the cell suspensions. The cell suspensions were serially diluted and seeded into six-well plates containing the culture medium, followed by a 6-day incubation period. Upon the emergence of macroscopic colonies in the culture dishes, the cultivation process was concluded. The supernatant was removed, and the cells were fixed with 4% paraformaldehyde (DF0135, Leagene Biotechnology Co., Ltd., China) for 20 min. Subsequently, staining with crystal violet staining solution (DZ0053, Leagene Biotechnology Co., Ltd., China) was carried out for 15 min. Ultimately, the clone formation rates were computed.

$$\text{Clone efficiency}=\left(\text{number of clones }/\text{ number of seeded cells}\right)\times 100$$

### 2.12 Transwell cell migration assay

CHMP4C-overexpressing MG63 cells, CHMP4C-silenced MG63 cells, and their respective empty vector control cells were adjusted to a density of 5×10^5^/mL cells/mL in serum-free medium and seeded in Transwell chambers (3,421, Corning Inc., USA). The lower chambers were loaded with DMEM medium containing 10% FBS and 1% streptomycin, and then placed in a cell culture incubator for 24 h. Following incubation, Transwell chambers were extracted, and the culture medium was aspirated. The cells were washed twice with calcium-free PBS, fixed with 4% paraformaldehyde for 20 min, and stained with 0.1% crystal violet for another 20 min. The upper layer of the non-migrated cells was gently wiped off with a wet cotton ball. After washing with PBS, cells from three randomly selected fields were counted, and the average was calculated under a 100x microscope (CKX3-SLP, OLYMPUS, Japan).

### 2.13 Animal grouping and model construction

Thirty-two male specific pathogen free (SPF)-grade BALB/C nude mice, approximately 6 weeks old, were obtained from Chongqing Ensiweier Biological Technology Co., Ltd., China. Mice were randomly divided into four groups (OE-NC, OE-CHMP4C, IN-NC, and IN-CHMP4C), each consisting of eight mice. The mice were acclimated in an animal facility at 25°C and a 12–12 h light-dark cycle for 1 week. MG63 cells overexpressing CHMP4C, MG63 cells with CHMP4C knockdown, and their corresponding control MG63 cells were digested with trypsin (S310KJ, Beyotime Institute of Biotechnology, China) to prepare cell suspensions at a cell density of 3×10^7^ cells/mL. The skin of the mice at the thigh root was disinfected and each mouse was subcutaneously injected with a cell suspension (0.5 mL). After 4 weeks, the mice were anesthetized and tumor images were observed and captured. Tumor dimensions were gauged, and euthanasia of the mice was carried out. The tumors were fully excised and weighed. Following that, RT-qPCR and immunofluorescence techniques were employed to assess the mRNA and protein expression levels. The animal study protocol was approved by the Ethics Committee of Affiliated Hospital of Qingdao University (QYFY WZLL 28188, 2023.11.06).

### 2.14 Statistical analysis

GraphPad Prism 8.0 (GraphPad Company, San Diego, CA, USA) was used for data analysis. The results are presented as mean ± standard deviation. Each experiment was performed at least three times. Independent sample t-tests were used for comparisons between two groups, and one-way analysis of variance (ANOVA) and Tukey’s *post hoc* test were used for comparisons among three or more groups. A *p*-value <0.05 was considered statistically significant.

## 3 Results

### 3.1 The selection and enrichment of DEGs

**FIGURE 1:**
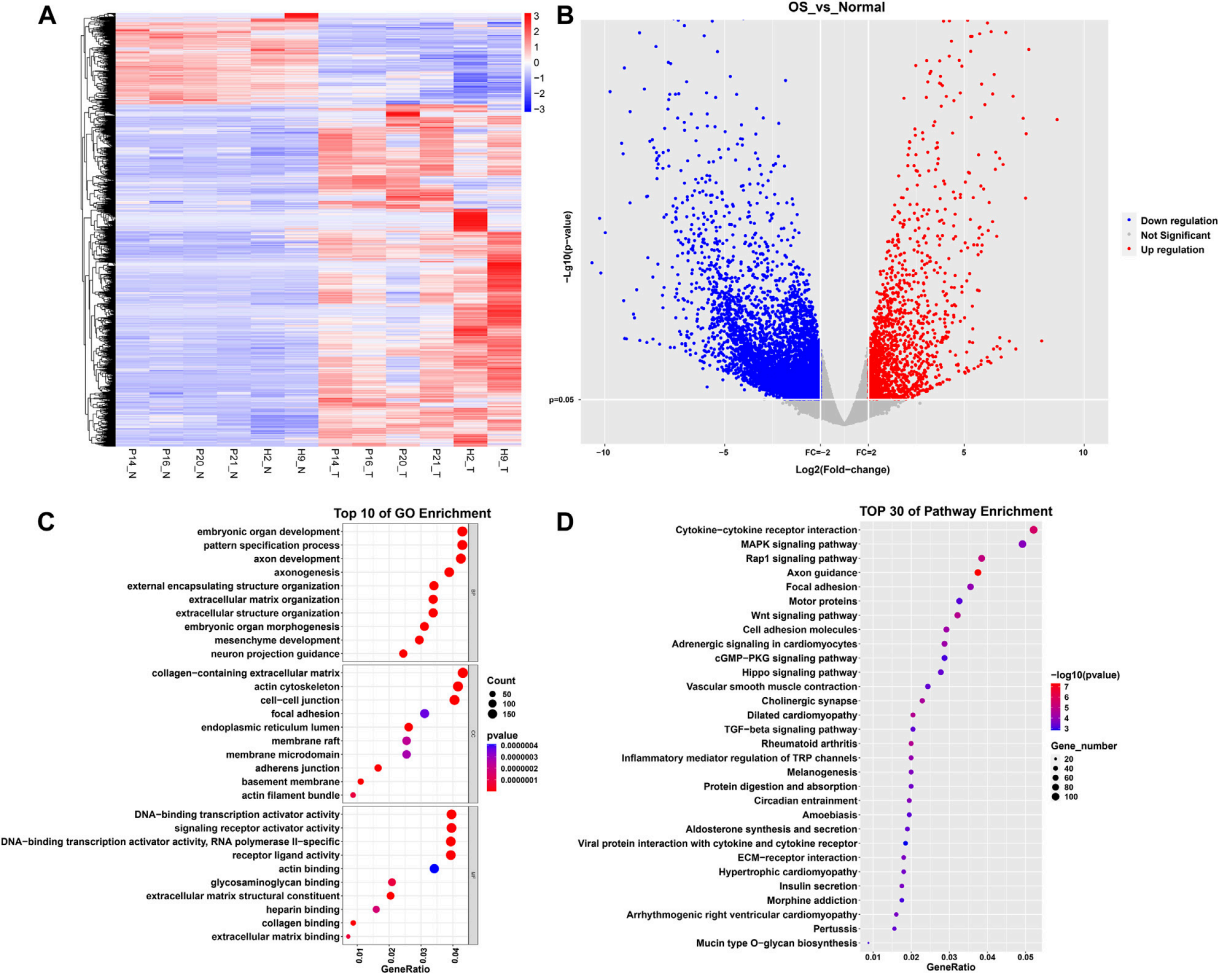
Differential gene expression analysis and enrichment analysis between osteosarcoma and healthy bone tissue. **(A)** Heatmap. **(B)** Volcano plot. **(C)** Top 10 biological processes, cellular components, and molecular functions sorted by *p*-values in GO analysis. **(D)** Top 30 pathways sorted by *p-*values in KEGG analysis.

A total of 7,189 DEGs were identified, with 1,511 DEGs upregulated in normal tissue samples and 5,678 DEGs downregulated. Figure 1A presents the heatmap generated from hierarchical clustering analysis. The volcano plot illustrates the differential gene expression between osteosarcoma tissue and normal tissue (Figure 1B). To unveil the potential biological functions of DEGs, we conducted GO and KEGG analyses. Those enriched biological processes in the GO analysis included embryonic organ development, pattern specification process, and axon development. Enriched cellular components comprised the collagen-containing extracellular matrix, actin cytoskeleton, and cell-cell junction. Additionally, enriched molecular functions encompassed DNA-binding transcriptional activator activity, RNA polymerase II-specific, DNA-binding transcription activator activity, receptor ligand activity, and signaling receptor activator activity (Figure 1C). KEGG analysis revealed enrichment in pathways such as axon guidance, cytokine-cytokine receptor interaction, Wnt signaling, Rap1 signaling, and Mitogen-Activated Protein Kinase (MAPK) signaling (Figure 1D).

### 3.2 Determination of key modules through WGCNA

**FIGURE 2:**
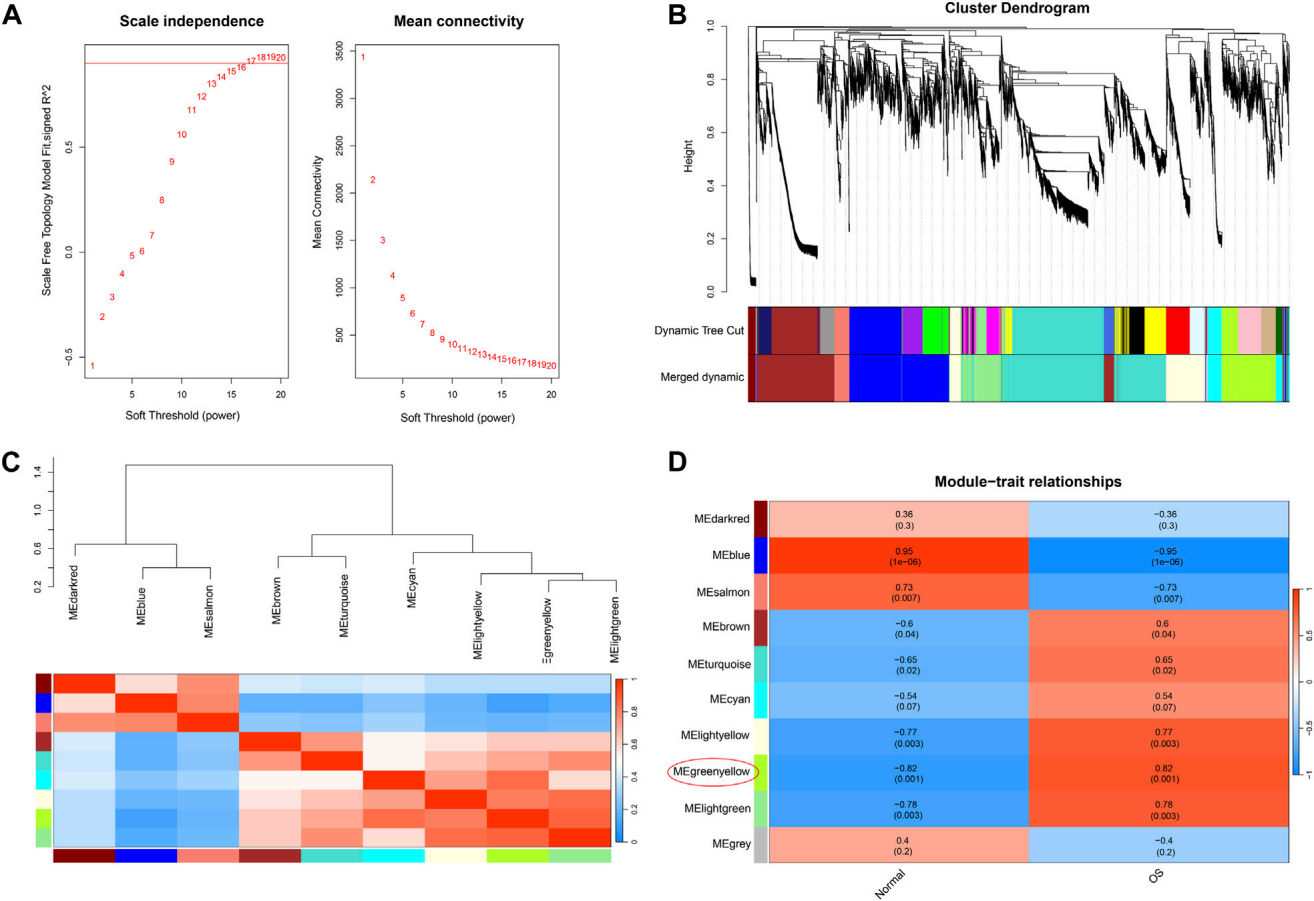
Weighted gene co-expression network analysis. **(A)** Confirmation of the soft threshold (β = 17) through scale independence and average connectivity calculations. **(B)** Hierarchical clustering tree displaying key modules composed of DEGs. **(C)** Module correlation analysis. **(D)** Selection of modules associated with osteosarcoma based on module-trait relationships.

**FIGURE 3:**
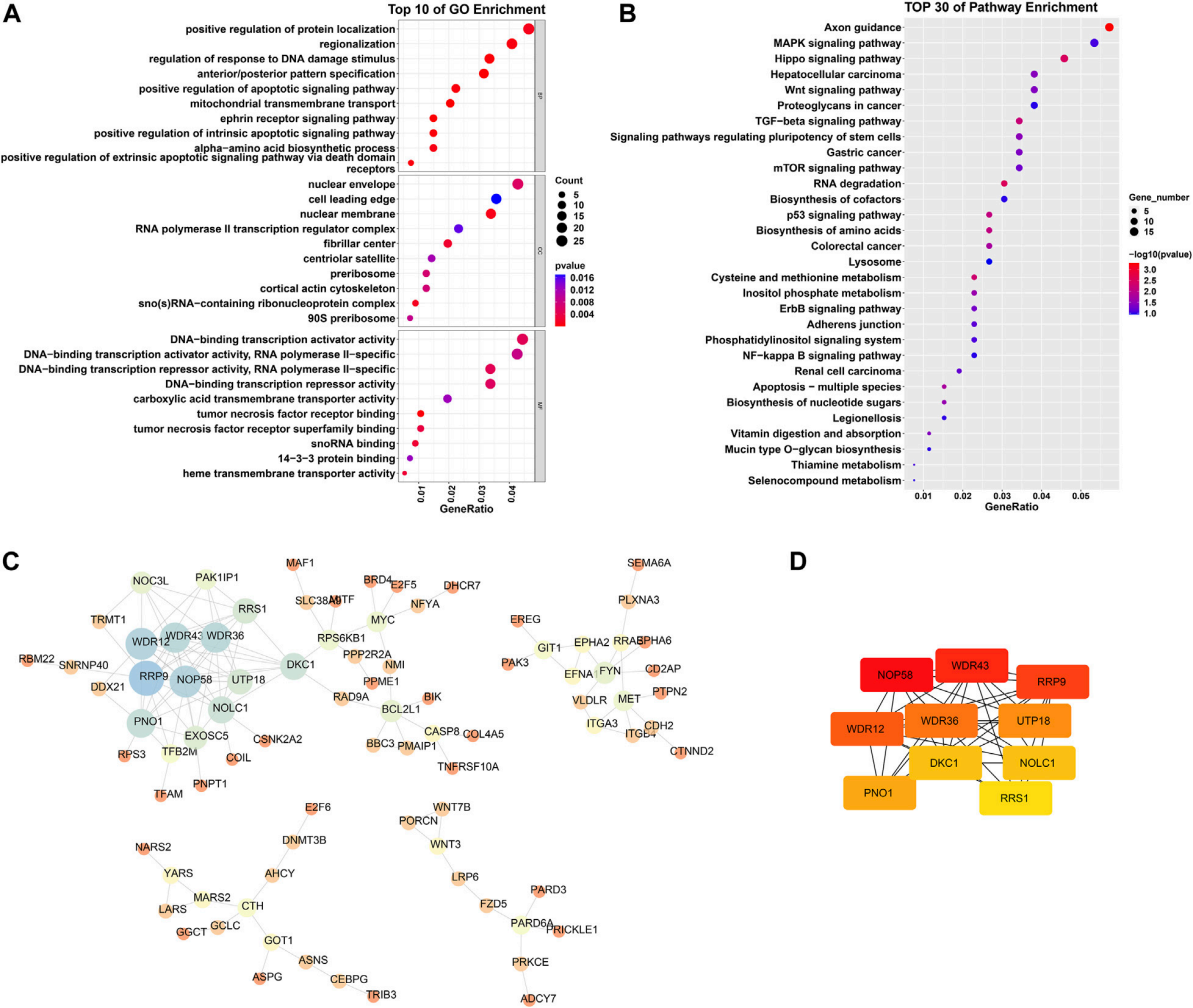
GO and KEGG enrichment analysis of DEGs in the greenyellow module, as well as PPI network analysis. **(A)** Top 10 biological processes, cellular components, and molecular functions ranked by *p*-values in GO analysis. **(B)** Top 30 pathways ranked by *p*-values in KEGG analysis. **(C)** PPI network analysis. Nodes of the network represent proteins; the size of each node represents the betweenness centrality of the node; node color represents the size of the degree, blue indicates a high degree, while red indicates a low degree. **(D)** Top 10 genes calculated by the MCC method. The greater the intensity of red, the higher the degree.

Through scale independence and average connectivity comparisons, we found that when the soft threshold was set to 17 for gene-to-gene connections (Figures 2A, B), we were able to construct a hierarchical clustering tree containing 10 key modules (Figure 2C). However, it is worth noting that genes within the gray module appeared to lack distinct functional similarities. To ascertain the significance of each module, we conducted correlation analysis between the obtained modules and clinical features. The results revealed a significant positive correlation between the green-yellow module and osteosarcoma (R = 0.82, *p* = 0.001) (Figure 2D), which encompassed 754 DEGs. To gain a deeper understanding of the biological functions of DEGs within the green–yellow module, we performed GO and KEGG enrichment analyses. GO functional enrichment encompasses biological processes, such as regulation of the response to DNA damage stimulus, regionalization, and positive regulation of protein localization. Concerning cellular components, enrichment was noted in the nuclear membrane, nuclear envelope, and cell leading edge. Molecular functions exhibited enrichment in DNA-binding transcriptional repressor activity, RNA polymerase II-specific DNA-binding transcription activator activity, DNA-binding transcription repressor activity, DNA-binding transcription activator activity, and RNA polymerase II-specific (Figure 3A). Furthermore, in the KEGG enrichment analysis, we identified associations with pathways such as axon guidance, Hippo signaling pathway, hepatocellular carcinoma, Wnt signaling pathway, and MAPK signaling pathway (Figure 3B).

### 3.3 PPI network analysis

We constructed and analyzed a protein-protein interaction network for the DEGs in the green–yellow module. We imported the DEG information into the STRING database and further analyzed the generated gene interaction network using Cytoscape software (Figure 3C). Utilizing the Maximal Clique Centrality (MCC) algorithm in the “Cytohubba” plugin, we identified the top 10 genes that exhibited the closest interactions within the gene network. These genes were Nucleolar Protein 58 (NOP58), WD Repeat Domain 43 (WDR43), Ribosomal Protein R9 (RPR9), WD Repeat Domain 12 (WDR12), WD Repeat Domain 36 (WDR36), U3 small nucleolar RNA-associated protein 18 (UTP18), Partner of NOB1 homolog (PNO1), Dyskerin Pseudouridine Synthase 1 (DKC1), Nucleolar and Coiled-body Phosphoprotein 1 (NOLC1), and Ribosomal RNA Processing 1 (RRS1) (Figure 3D). The darker color signifies a more significant role of these genes in the occurrence and development of osteosarcoma.

### 3.4 Construction of osteosarcoma patient prognosis assessment risk model and survival analysis

**FIGURE 4:**
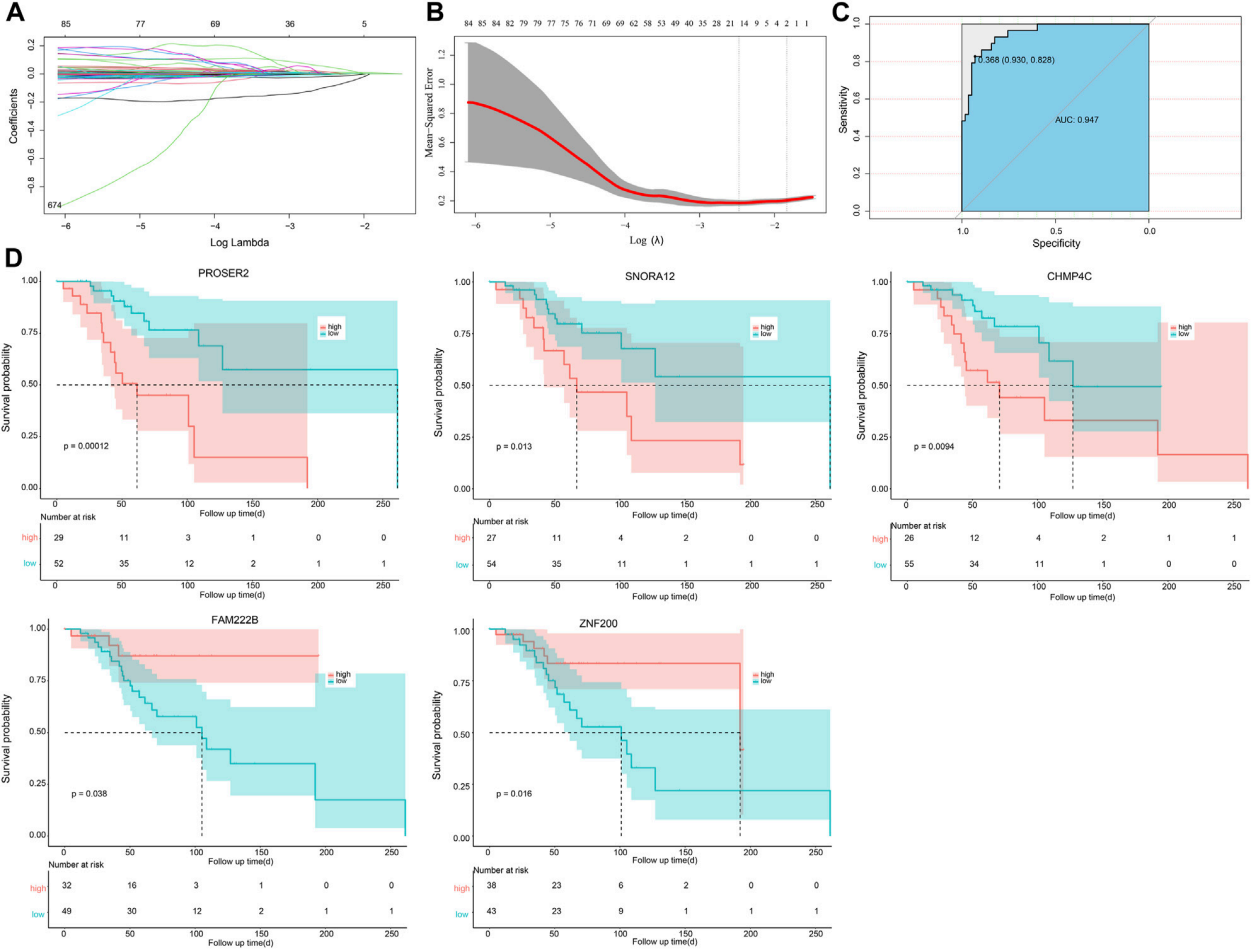
Risk model and survival analysis for prognostic evaluation of OS Patients. **(A,B)** LASSO selection analysis plots of OS-related genes within the greenyellow module. **(C)** The ROC curve. **(D)** Kaplan-Meier survival analysis results, showing gene survival analysis curves significantly associated with the prognosis of OS patients (including CHMP4C, FAM222B, PROSER2, SNORA12, ZNF200).

**FIGURE 5:**
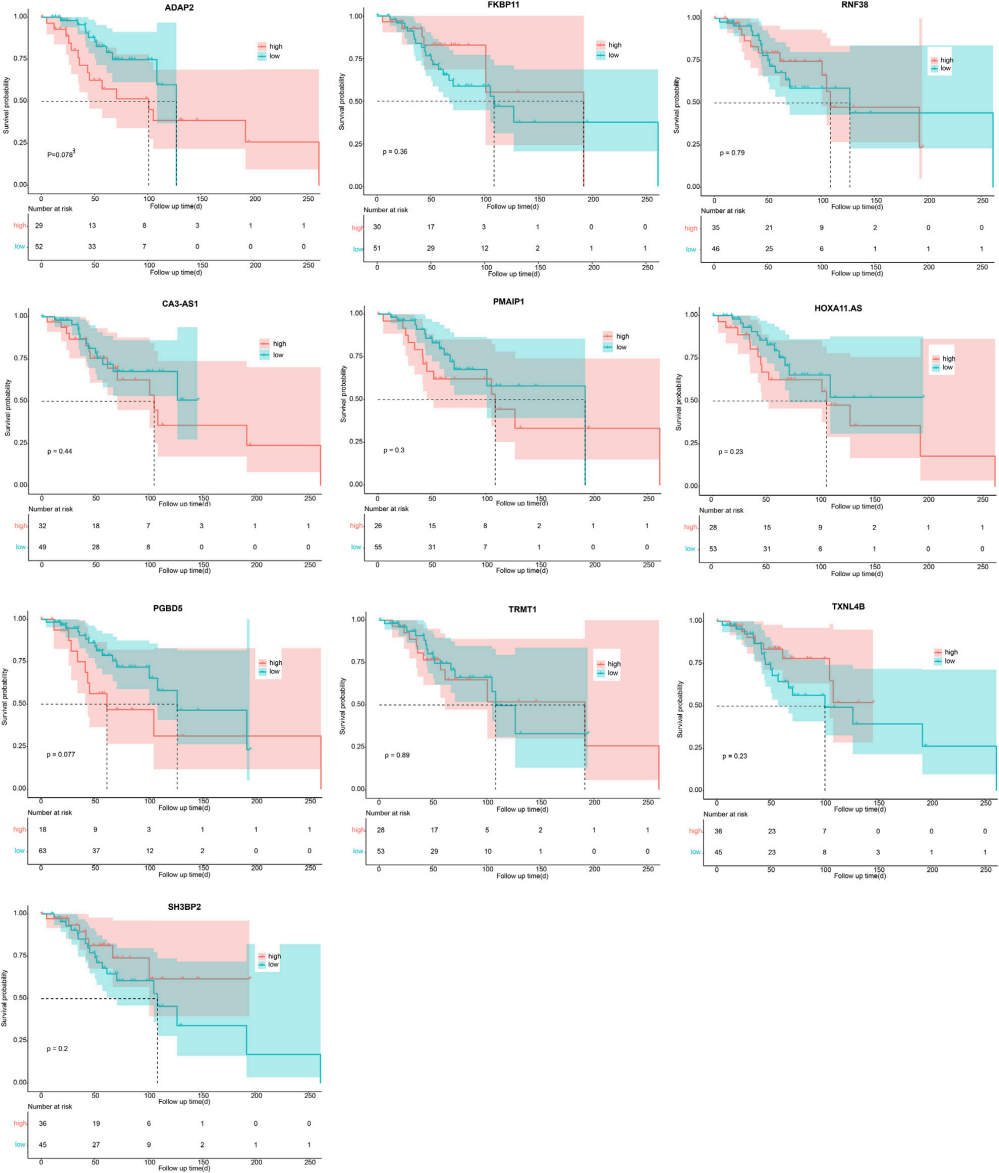
Survival analysis of 10 genes in OS patients (ADAP2, FKBP11, RNF38, CA3-AS1, PMAIP1, HOXA11. AS, PGBD5, TRMT1, TXNL4B, SH3BP2) via Kaplan-Meier method.

The optimal λ that produces the smallest classification error was identified in the LASSO model through 10-fold cross-validation. Subsequently, 15 hub genes with non-zero coefficients were identified (ArfGAP with dual PH domains 2, ADAP2; Carbonic Anhydrase III-Antisense RNA 1, CA3-AS1; Charged Multivesicular Body Protein 4C, CHMP4C; Family with sequence similarity 222 member B, FAM222B; FK506 Binding Protein 11, FKBP11; HOXA11 antisense RNA, HOXA11. AS; PiggyBac transposable element derived 5, PGBD5; Phorbol-12-Myristate-13-Acetate-Induced Protein 1, PMAIP1; Proline and serine rich 2, PROSER2; Ring Finger Protein 38, RNF38; SH3 Domain Binding Protein 2, SH3BP2; Small Nucleolar RNA-H/ACA Box 12, SNORA12; tRNA Methyltransferase 1 Homolog, TRMT1; Thioredoxin Like 4B, TXNL4B; Zinc Finger Protein 200, ZNF200) (as shown in Figures 4A, B). To enhance the scrutiny of the risk model’s accuracy in prognosis analysis, we generated ROC curves for the predictive model. The calculated AUC was 0.947, indicating a substantial predictive capability (Figure 4C). This suggests that the risk prognosis model has a high predictive accuracy. To affirm the association between the expression of these 15 genes and OS patients, Kaplan-Meier survival analysis was employed. Patients were stratified into high- and low-expression groups, determined by the median gene expression values. Our outcomes revealed statistically significant differences in survival rates between low- and high-expression groups for the CHMP4C, FAM222B, PROSER2, SNORA12, and ZNF200 genes (*p* < 0.05) (Figure 4D). Notably, CHMP4C displayed the most substantial difference (*p* = 0.0094), signifying a robust correlation between CHMP4C expression and the prognosis of OS patients. Individuals in the low-expression group of CHMP4C exhibited a more favorable prognosis with higher survival rates (*p* < 0.05). The survival curves for the other nine genes are shown in Figure 5.

### 3.5 Expression of CHMP4C and downstream pathway genes in various osteosarcoma cell lines

**FIGURE 6:**
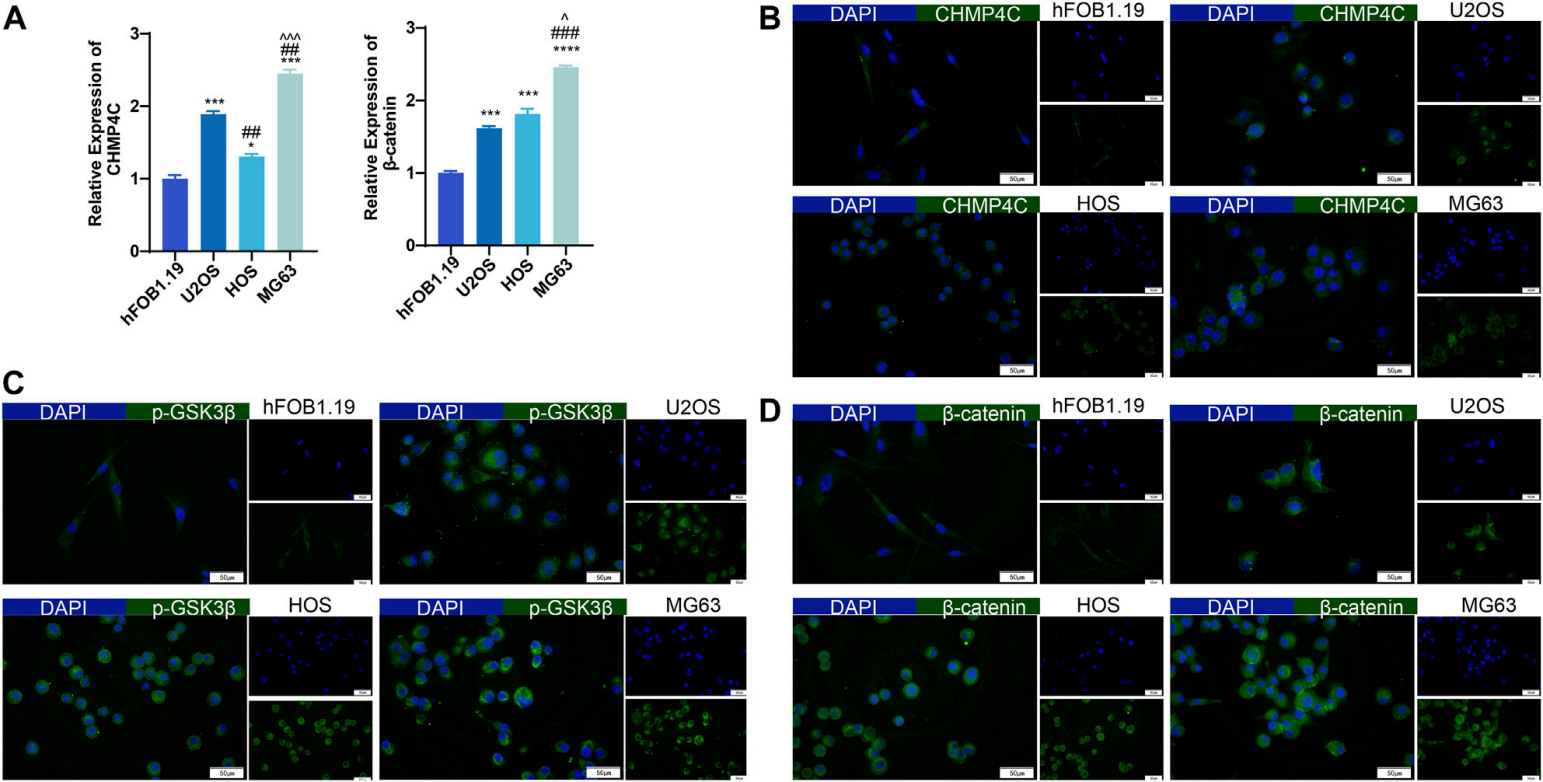
Expression of CHMP4C and β-catenin in hFOB1.19, U2OS, HOS, and MG63 cells. **(A)** RT-qPCR analysis of CHMP4C and β-catenin mRNA expression. **(B–D)**: Immunofluorescence staining to detect the expression of CHMP4C, p-GSK3β, and β-catenin proteins. The Merge image is on the left side, DAPI (blue) is above on the right side, and FITC (green) is below on the right side. ^*^ *p* < 0.05, ^***^ *p* < 0.001, ^****^ *p* < 0.0001 compared with hFOB1..19 group. ^##^ *p* < 0.01, ^###^ *p* < 0.001 compared with U2OS group. ^^^ *p* < 0.05, ^^^^^ *p* < 0.001 compared with HOS group.

In this study, we conducted an analysis of CHMP4C and the related proteins GSK3β, p-GSK3β, and β-catenin. CHMP4C is a protein associated with the endocytic pathway and closely linked to processes such as cell division, endocytosis, and signal transduction (Zhang et al., 2022). Research has indicated a significant association between the GSK3β/β-catenin signaling pathway and osteosarcoma proliferation (Ruan and Zhao, 2018). In the RT-qPCR analysis, we observed distinct expression patterns of CHMP4C and β-catenin across the four OS cell lines. Specifically, CHMP4C mRNA expressions were markedly elevated in U2OS, HOS, and MG63 cells compared to hFOB1.19 cells (*p* < 0.01), with MG63 cells exhibiting the highest expression among them. Conversely, β-catenin mRNA expression levels increased sequentially in hFOB1.19, U2OS, HOS, and MG63 cells, with statistical significance compared to hFOB1.19 (*p* < 0.01) (Figure 6A). Our outcomes of immunofluorescence staining revealed notable disparities in the protein expression levels of CHMP4C, p-GSK3β, and β-catenin among the U2OS, HOS, and MG63 osteosarcoma cells, with significantly higher expression observed in these cells compared to hFOB1.19 cells (Figures 6B−D).

### 3.6 Impacts of CHMP4C overexpression and interference on the expression of GSK3β, p-GSK3β, and β-catenin in MG63 cells

**FIGURE 7:**
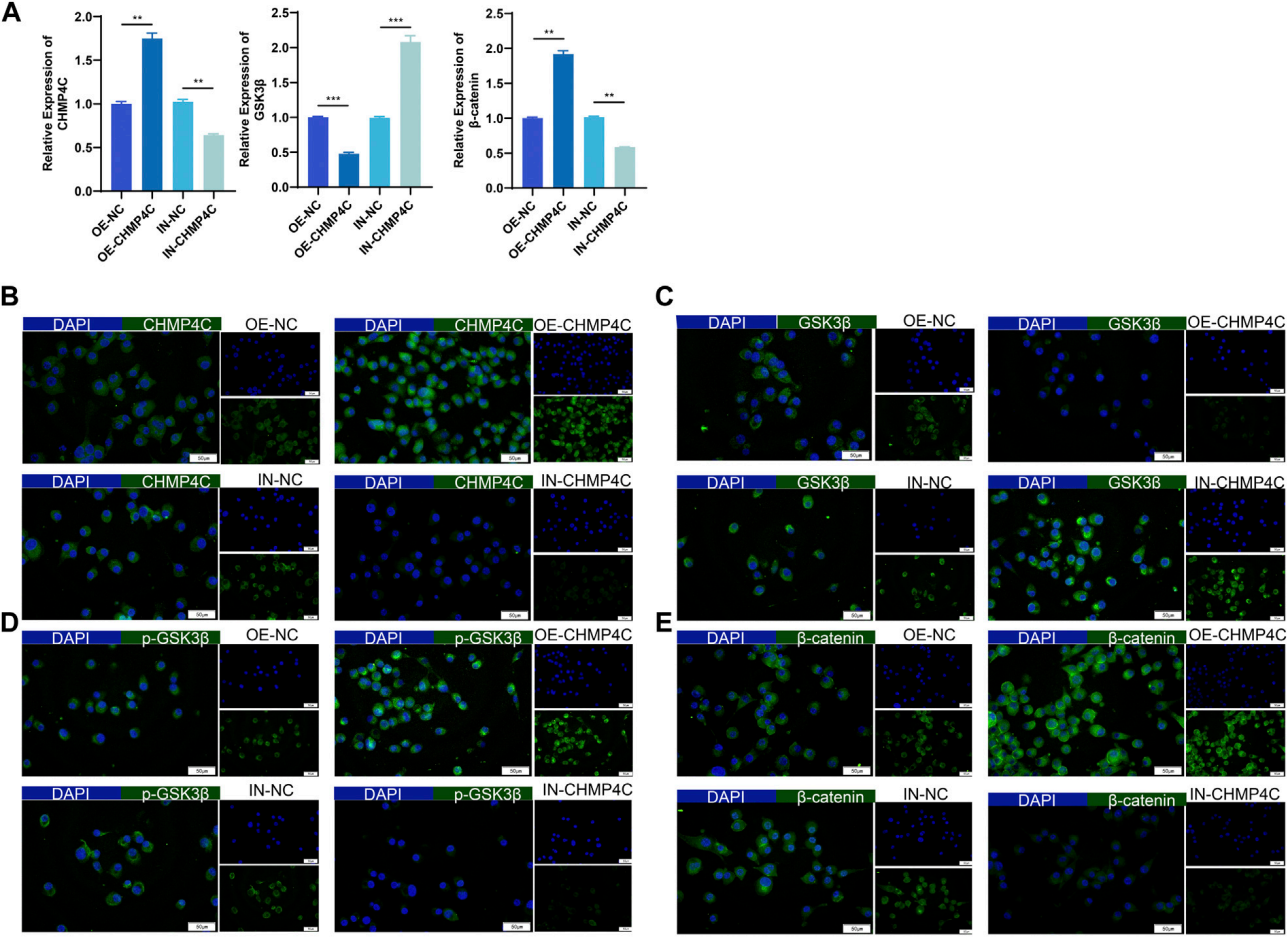
Impacts of overexpression and knockdown of CHMP4C on the expression of GSK3β, p-GSK3β, and β-catenin in MG63 cells. **(A)** RT-qPCR analysis of CHMP4C, GSK3β, and β-catenin mRNA expression in MG63 cells. **(B–E)**: Immunofluorescence staining to assess the expression of CHMP4C, GSK3β, p-GSK3β, and β-catenin proteins in MG63 cells. The Merge image is on the left side, DAPI (blue) is above on the right side, and FITC (green) is below on the right side. ^**^ *p* < 0.01, ^***^ *p* < 0.001 compared with OE-NC / IN-NC group.

In our exploration of the biological function of CHMP4C in OS cells, MG63 cells were transfected with a synthetic CHMP4C over-expression vector, CHMP4C interference lentiviral vector, and an empty vector for validation. RT-qPCR results revealed that, in comparison to empty vector group, CHMP4C over-expression notably augmented the mRNA expressions of CHMP4C and β-catenin in MG63 cells (*p* < 0.0001) and concurrently diminished the expressions of GSK3β (*p* < 0.001). Conversely, CHMP4C knockdown significantly repressed the expression of CHMP4C and β-catenin in MG63 cells (*p* < 0.001) while elevating GSK3β expression (*p* < 0.0001) (Figure 7A). Immunofluorescence staining further validated these findings. CHMP4C over-expression notably heightened the protein expression of CHMP4C (Figure 7B), p-GSK3β (Figure 7D), and β-catenin (Figure 7E) in MG63 cells, while concurrently reducing GSK3β expression (Figure 7C). In contrast, CHMP4C knockdown greatly curtailed the protein expression of CHMP4C (Figure 7B), p-GSK3β (Figure 7D), and β-catenin (Figure 7E) in MG63 cells, while elevating GSK3β expression (Figure 7C).

### 3.7 Impacts of CHMP4C overexpression and CHMP4C interference on the proliferation and migration of OS cells

**FIGURE 8:**
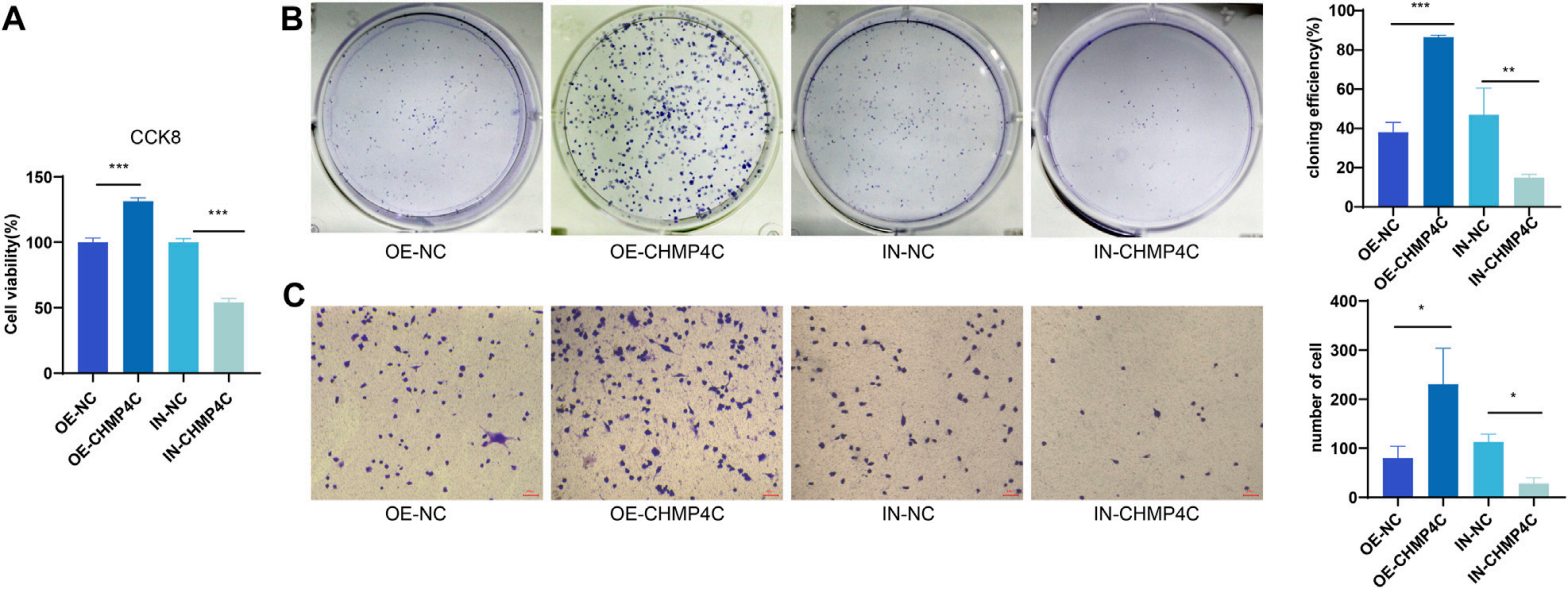
Assessment of the impacts of CHMP4C overexpression and knockdown on the proliferation and migration of OS Cells. **(A)** CCK-8 assay for MG63 cell proliferation. **(B)** Colony formation assay for quantifying the number and efficiency of MG63 cell colonies. **(C)** Transwell assay for assessing the migration of MG63 cells. ^*^ *p* < 0.05, ^**^ *p* < 0.01, and ^***^ *p* < 0.001 compared with OE-NC / IN-NC group.

Our CCK-8 assay outcomes demonstrated that, in comparison to the control group, CHMP4C over-expression significantly heightened the proliferation of MG63 cells, whereas interference with CHMP4C expression markedly curtailed MG63 cell proliferation (*p* < 0.0001) (Figure 8A). Findings from the colony formation assay revealed that the number of MG63 cells and cloning efficiency were notably elevated in the CHMP4C over-expression group in contrast to control group (*p* < 0.001) (Figure 8B). Conversely, the number of MG63 cells and cloning efficiency were substantially reduced in CHMP4C interference group as compared to control group. In Transwell migration assays, compared to control group, CHMP4C over-expression resulted in a significant augmentation in the number of migrating MG63 cells (*p* < 0.0001), whereas interference with CHMP4C expression led to a notable reduction in the number of migrating MG63 cells (*p* < 0.001) (Figure 8C).

### 3.8 Impacts of CHMP4C overexpression and interference on mouse osteosarcoma growth and downstream pathway gene expression

**FIGURE 9:**
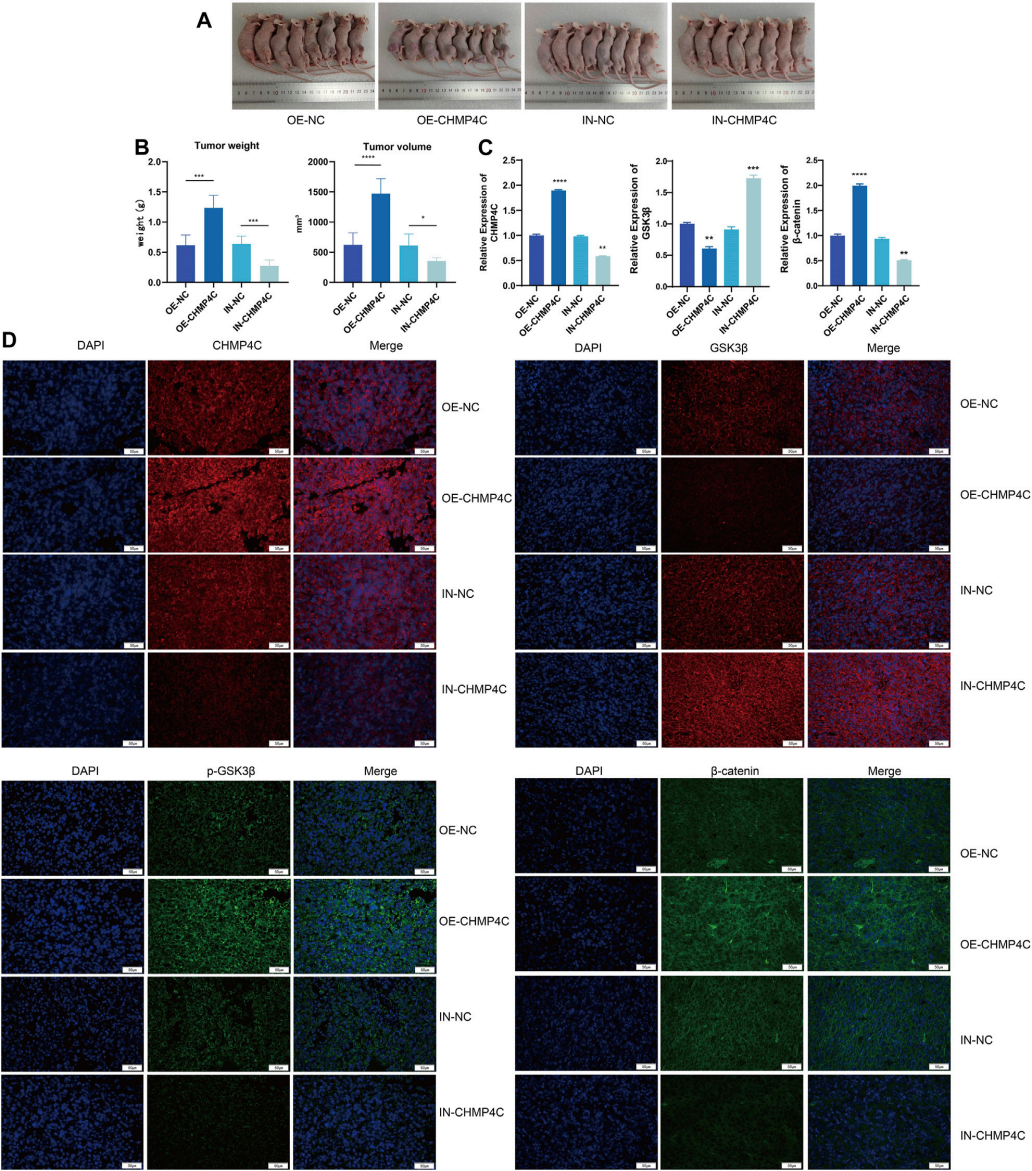
Impacts of CHMP4C overexpression and knockdown on the growth of nude mouse OS and the expression of downstream pathway genes. **(A,B)**: Effects of CHMP4C overexpression and knockdown on the weight and volume of nude mouse OS. **(C)** RT-qPCR analysis of mRNA expression levels of CHMP4C, GSK3β, and β-catenin in tumor tissues. **(D)** Immunofluorescence staining analysis of protein expression levels of CHMP4C, GSK3β, p-GSK3β, and β-catenin in tumor tissues. ^*^ *p* < 0.05, ^**^ *p* < 0.01, ^***^ *p* < 0.001, and ^****^ *p* < 0.0001 compared with OE-NC / IN-NC group.

We further investigated the tumor occurrence through subcutaneous tumor experiments in nude mice. Compared to the empty vector group, the CHMP4C overexpression group showed a significant increase in tumor weight and volume (*p* < 0.0001), while the CHMP4C knockdown group exhibited a significant decrease in tumor weight (*p* < 0.001), with no significant difference in tumor volume (*p* > 0.05) (Figures 9A, B). RT-qPCR analysis of mRNA expression in tumor tissues revealed that compared to the empty vector group, CHMP4C overexpression significantly increased the expression of CHMP4C and β-catenin in mouse tumor tissues (*p* < 0.0001) and significantly decreased GSK3β expression (*p* < 0.001). Conversely, interference with CHMP4C expression significantly reduced the expression of CHMP4C and β-catenin, and increased GSK3β expression (*p* < 0.0001) (Figure 9C). Our outcomes of immunofluorescence staining revealed that the protein expressions of CHMP4C, p-GSK3β, and β-catenin were notably elevated in CHMP4C over-expression group compared to empty vector group. Conversely, the GSK3β protein expression was significantly lower in CHMP4C over-expression group. In contrast, in CHMP4C knockdown group, the protein expressions of CHMP4C, p-GSK3β, and β-catenin were markedly lower than those in empty vector group, while the GSK3β protein expression was significantly higher (Figure 9D).

## 4 Discussion

Osteosarcoma (OS) is recognized as one of the most aggressive bone tumors, predominantly affecting children and adolescents. Significant risk factors associated with the progression of OS include age, gender, height, exposure to alkylating agents, bone turnover, chromosomal abnormalities such as hereditary retinoblastoma, ionizing radiation, and Paget’s disease (Kanematsu et al., 2010; O'Donovan et al., 2011). OS occurs in any bone throughout the body, although it predominantly occurs in the metaphysis of long bones. The rapid progression and metastasis of OS pose significant challenges for its treatment. Approximately 20% of patients present with lung metastasis at the time of diagnosis, and the majority of recurrent OS cases involve lung metastasis (Pastorino et al., 2023). Current standard treatments for OS include neoadjuvant chemotherapy, surgical resection of the primary tumor, and adjuvant chemotherapy. However, the prognosis for metastatic or recurrent OS has not significantly improved (He et al., 2022). Traditional clinical markers have limited predictive capabilities because the molecular mechanisms underlying OS are unclear. Research has shown that the pathogenesis of OS is closely related to gene expression (Lin et al., 2017). Therefore, it is essential to identify new diagnostic and prognostic biomarkers for more accurate diagnosis and prediction of OS. Transcriptomic data analysis using bioinformatics enables the identification of differentially expressed genes (DEGs) between tumor and normal tissues, offering promising molecular markers for various cancers (Wu et al., 2017; Solinas et al., 2019). Hence, employing high-throughput methods to explore effective molecular markers and potential therapeutic targets for OS patients is of utmost significance. In this study, mRNA expression data from OS patients were extracted from the GEO database and analyzed, leading to the identification of 7,189 DEGs. Among these, 1,511 were upregulated, and 5,678 were downregulated in OS tissues compared to healthy bone tissues. Through a more comprehensive analysis via WGCNA, the green–yellow module was pinpointed, comprising 754 DEGs highly associated with OS. Functional enrichment analysis unveiled the involvement of these DEGs in processes such as the regulation of the response to DNA damage stimulus, regionalization, positive regulation of protein localization, nuclear membrane, nuclear envelope, cell leading edge, RNA polymerase II-specific, DNA-binding transcription activator activity, and DNA-binding transcription repressor activity. Furthermore, KEGG pathway enrichment analysis indicated the signal participation of these DEGs in pathways including axon guidance, the Hippo signaling pathway, hepatocellular carcinoma, the Wnt signaling pathway, and the MAPK signaling pathway.

Recent research has shown that the Wnt signaling pathway plays a crucial role in the growth, proliferation, invasion, and metastasis of OS. It also contributes to OS drug resistance and regeneration, making it a significant player in the development of OS (Singla et al., 2020). The Wnt signaling pathway is a highly conserved growth factor family pathway with biological functions that regulate tumor cell proliferation, differentiation, apoptosis, and invasion. It comprises four distinct pathways: Wnt/β-catenin pathway, Wnt/Ca^2+^ pathway, Wnt/planar cell polarity pathway, and Wnt/protein kinase A pathway (Wang et al., 2022). Research has confirmed a close association between the Wnt/β-catenin pathway and OS development (Zou et al., 2017). It is believed that the Wnt/β-catenin signaling pathway is activated in OS, and inhibition of this pathway can suppress the proliferation and malignant behavior of OS cells (Xu et al., 2020). Implementing targeted interventions at the upstream level of Wnt proteins to impede the entry of downstream factors into the β-catenin pathway could potentially serve as a preventive measure against the development of OS (Huang et al., 2022). Nevertheless, conflicting findings have been documented, indicating instances where the activation of the Wnt/β-catenin pathway facilitated the osteogenic differentiation of OS cells and concurrently impeded their proliferation. In this scenario, the inactivation of the Wnt/β-catenin pathway might potentially contribute to the development of OS (Cai et al., 2010). GSK3β functions as a crucial negative regulator within the Wnt/β-catenin signaling pathway. Upon activation, GSK3β actively facilitates the phosphorylation of β-catenin, initiating its degradation through ubiquitination. Reduction of GSK3β phosphorylation at serine nine leads to its activation, effectively suppressing the Wnt/β-catenin signaling pathway and thereby impeding the progression of OS (Lu et al., 2023). Therefore, gaining a comprehensive understanding of the mechanisms by which the Wnt signaling pathway functions in OS is of great significance for inhibiting its occurrence and progression.

We further established a prognostic risk model for OS patients through LASSO analysis by selecting 15 core genes (ADAP2, CA3-AS1, CHMP4C, FAM222B, FKBP11, HOXA11. AS, PGBD5, PMAIP1, PROSER2, RNF38, SH3BP2, SNORA12, TRMT1, TXNL4B, and ZNF200). ROC curve analysis showed that the predictive model exhibited good accuracy (AUC = 0.947). Survival analysis confirmed the prognostic relevance of these core genes in patients with OS, with CHMP4C being the most significant (*p* = 0.0094). Patients with low CHMP4C expression had better prognoses, higher survival rates, and longer survival periods. Therefore, CHMP4C is considered to be the most crucial gene contributing to OS formation. CHMP4C plays a role in cell division by preventing accumulation of DNA damage through delayed abscission (Li et al., 2015). The polymorphism of CHMP4C increases susceptibility to cancer and may promote genomic instability, thereby inducing cancer (Liu et al., 2021). CHMP4C upregulation has been confirmed in cervical cancer tissues, promoting cell proliferation, migration, and invasion (Lin et al., 2020). Silencing CHMP4C can enhance the sensitivity of lung cancer cells to radiation by delaying the S phase of the cell cycle (Carlton et al., 2012). Additionally, Liu et al. found that CHMP4C may be an effective method for the prevention and treatment of lung squamous cell carcinoma (LUSC), and that CHMP4C is overexpressed in LUSC, and its decreased expression leads to an abnormal cell cycle transition in LUSC (Liu et al., 2021). Similar to our results, we found that CHMP4C, p-GSK3β, and β-catenin were significantly overexpressed in U2OS, HOS, and MG63 OS cells compared to hFOB1.19 cells, with MG63 cells showing the highest expression levels. There have been reports suggesting that CARM1 may interact with PELP1, enhance GSK3β phosphorylation, further promote β-catenin signaling, activate the pGSK3β/β-catenin signaling pathway, and facilitate OS cell proliferation (Li et al., 2017). To substantiate the influence of CHMP4C on OS, we conducted both over-expression and silencing experiments in MG63 OS cells. The outcomes demonstrated that CHMP4C over-expression significantly suppressed the expression of GSK3β in MG63 cells, while markedly elevating the levels of CHMP4C, pGSK3β, and β-catenin. Additionally, the over-expression of CHMP4C notably enhanced the proliferation and migration of MG63 cells. Conversely, interference with CHMP4C yielded opposite effects, aligning with the observations reported by Zhang et al. (2022). In addition, we validated the *in vivo* role of CHMP4C. Over-expression of CHMP4C drastically increased the expression of pGSK3β and β-catenin in xenografted OS in nude mice, while signally decreasing the expression of GSK3β. This resulted in an obvious augmentation in the growth of nude mouse OSs. Conversely, interference with CHMP4C produced the opposite effects. Our study implied that CHMP4C may activate the Wnt/β-catenin signaling pathway by enhancing the phosphorylation of GSK3β, thereby fostering the growth and development of OS. Therefore, CHMP4C may serve as a target gene for the onset and progression of OS and represent a potential therapeutic target.

**FIGURE 10:**
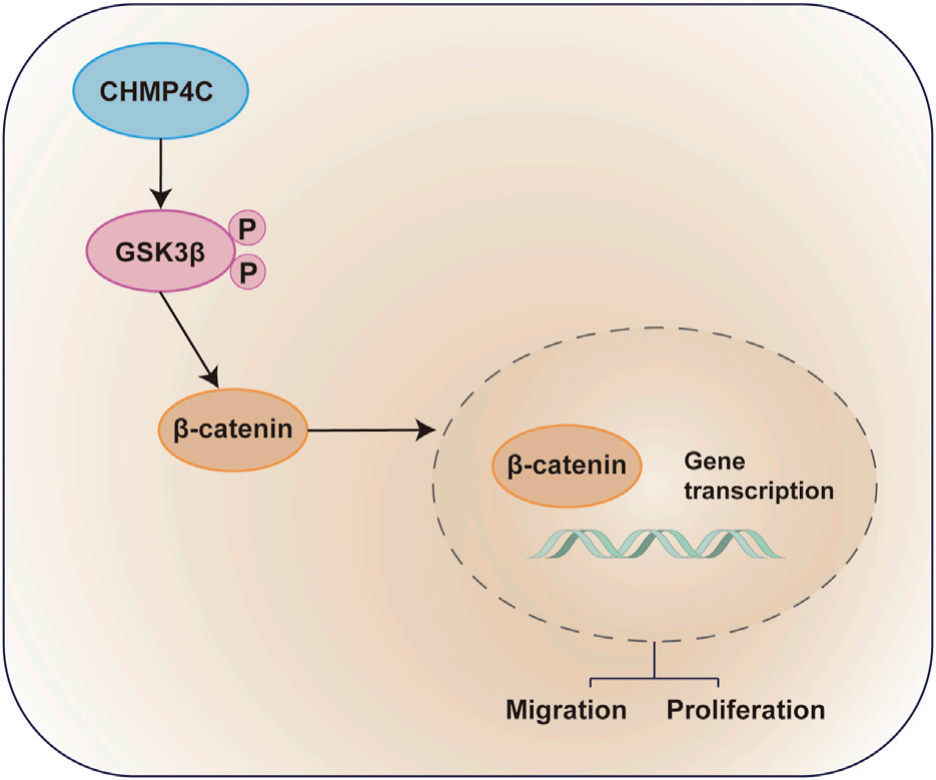
Diagram of the Molecular Mechanisms underlying CHMP4C CHMP4C within OS cells. CHMP4C inhibits the expression of GSK3β in OS cells, while upregulating the expression of pGSK3β and β-catenin. The phosphorylation of GSK3β further promotes β-catenin signaling transduction, activating the pGSK3β/β-catenin signaling pathway and promoting the proliferation and migration of OS cells.

In conclusion, this study pinpointed CHMP4C as a promising prognostic biomarker for OS. Through its ability to heighten the phosphorylation of GSK3β and activate the Wnt/β-catenin signaling pathway, CHMP4C plays a pivotal role in fostering the invasion and metastasis of osteosarcoma (Figure 10). These discoveries introduce novel biomarkers and therapeutic targets, providing insights for the diagnosis and treatment of osteosarcoma.

## Data Availability

The original contributions presented in the study are included in the article/Supplementary Material, further inquiries can be directed to the corresponding author.
